# Rotation of the second cervical vertebra in pediatric patient

**DOI:** 10.1590/S1679-45082016AI3631

**Published:** 2016

**Authors:** Priscila Dias Peyneau, Gina Delia Roque-Torres, Luiz Roberto Godolfim, Eliana Dantas da Costa, Solange Maria de Almeida, Gláucia Maria Bovi Ambrosano

**Affiliations:** 1 Faculdade de Odontologia de Piracicaba, Universidade Estadual de Campinas, Piracicaba, SP, Brazil.; 2 Universidade Federal do Rio Grande do Sul, Porto Alegre, RS, Brazil.

Rotatory instability is characterized by the rotation between two vertebral bodies, and it constitutes the most common cause of torticollis in children.^(1-5)^ This prevalence occurs because of specific anatomic characteristics of childhood, such as disproportion between head-neck, underdeveloped cervical musculature, laxity of the joint capsule, ligament elasticity and horizontal shape of the articular facets between atlas and axis vertebrae.^(3-7)^ This condition can occur due to inflammation/infection^(2,6,7)^ or trauma,^(1,2,4,6)^ or because of neurogenic or idiopathic origin.^(2)^

The diagnosis includes clinical and imaging exam.^(7,8)^ Among imaging exams of bone tissue, radiographies in anteroposterior and lateral projections are of limited use because they do not enable a precise visualization of this alteration, due to difficulties in positioning patients (head offset or source of X-rays, and overlap of structures), leading to radiographic interpretation challenges.^(1,6,7)^ Computed tomography is considered the gold standard procedure.^(1,6,7)^ Images of tridimensional reconstruction provides a global visualization of rotation, therefore helping to establish the diagnosis.^(2,3,7)^ In addition, the magnetic resonance image can also be requested to evaluate the risk of vascular-nervous bundle compromising and injuries of the ligaments adjacent to vertebrae.^(1,5-7)^

**Figure 1:**
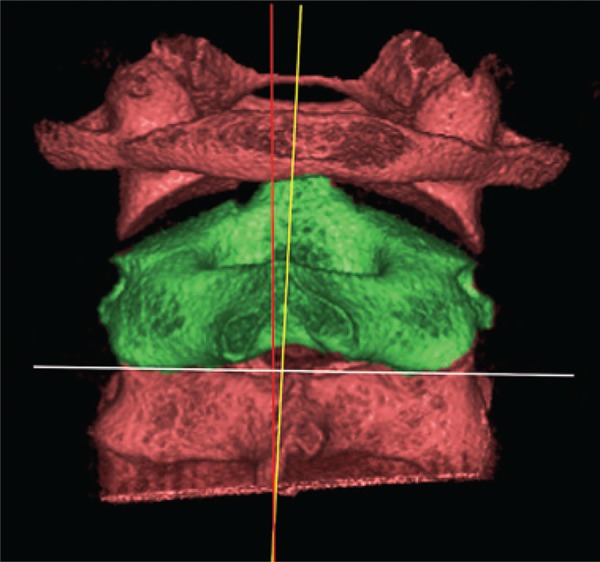
Tridimensional reconstruction of first three cervical vertebrae (posterior view). Rotation of the second cervical vertebra can be observed (green). The yellow line highlights the median sagittal plane; red line represents the rotation of second vertebra; white line indicates latero-lateral inclination of the second vertebra

A 12-year-old boy was referred to our radiologic clinic to undergo a cone beam computed tomography for orthodontic purposes. We carried out a tridimensional, axial, coronal and sagittal reconstruction (Figures 1 [Fig f02] [Fig f03] [Fig f04] to 5). During imaging assessment, we observed 5° of rotation of the second cervical vertebra in relation to medium line and a space between atlanto-axial vertebrae of 5.1mm (right side) and 7.6mm (left side) (Figure 5). In anamnesis, the patient reported trauma experienced 1 year earlier and, after the incident, presence of constant torticollis.

**Figure 2 f02:**
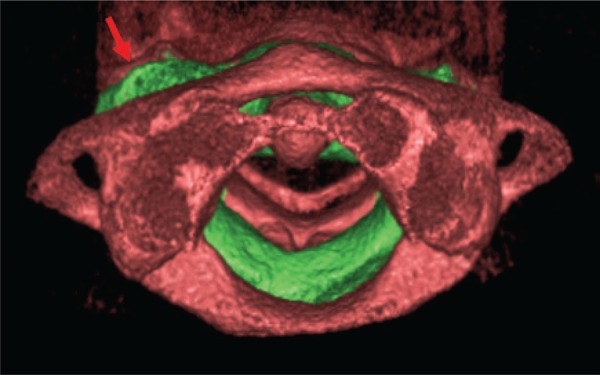
Tridimensional reconstruction of first three cervical vertebrae (transversal view). A rotation of the second vertebra is observed (green). Red arrow indicates the rotation of the second vertebra

**Figure 3 f03:**
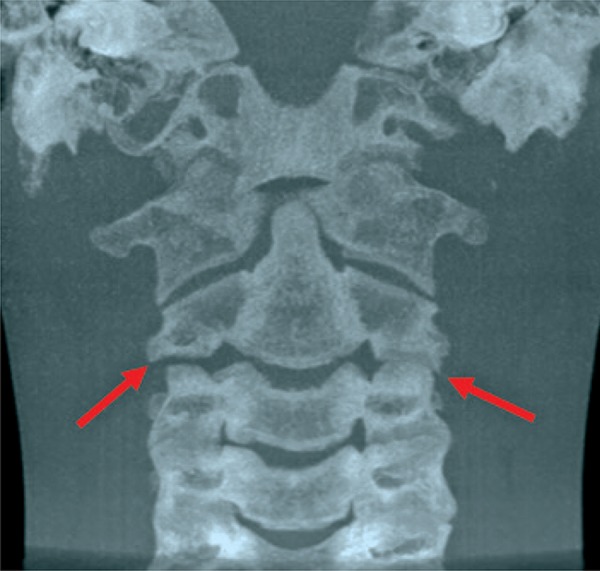
Coronal reconstruction of cervical vertebrae. Red arrows indicating latero-lateral inclination of the second vertebra

**Figure 4 f04:**
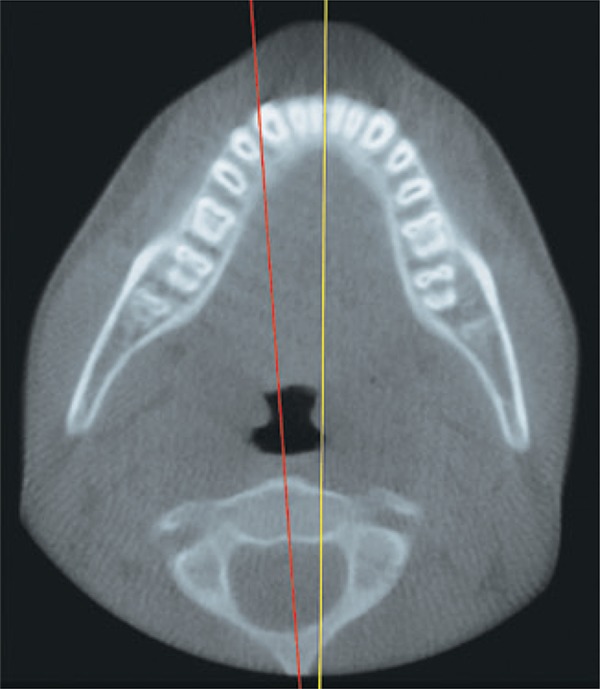
Axial reconstruction. Yellow line showing medium sagittal plan; Red line indicating rotation of the second cervical vertebra

**Figure 5:**
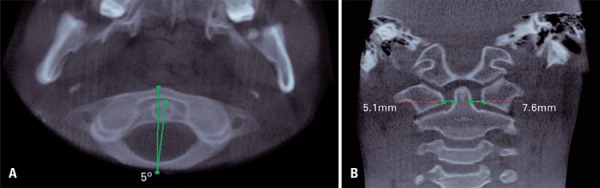
Cone beam computed tomography showing 5º of axial rotation in relation to medium line (A), atlanto-axil space of 5.1 and 7.6mm (B)

Correct diagnosis is crucial for adequate management. Treatment can be conservative using immobilization,^(3-5,7,9)^ traction or manual reduction,^(3,4,7,9)^ with the use of analgesic,^(3)^ physiotherapy^(1,9)^ or surgery.^(3,7,9)^
